# Bioanalytical Method Validations of Three Alpha1-Antitrypsin Measurement Methods Required for Clinical Sample Analysis

**DOI:** 10.3390/ph18081165

**Published:** 2025-08-06

**Authors:** Andrea Engelmaier, Martin Zimmermann, Harald A. Butterweck, Alfred Weber

**Affiliations:** 1 Pharmaceutical Science, Baxalta Innovations GmbH, Part of Takeda, 1220 Vienna, Austria; andrea.engelmaier@takeda.com; 2 Plasma Derived Therapies R&D, Baxalta Innovations GmbH, Part of Takeda, 1220 Vienna, Austria; martin.zimmermann@takeda.com (M.Z.); harald.butterweck@takeda.com (H.A.B.)

**Keywords:** α_1_-proteinase inhibitor, α_1_-anitrypsin, clinical sample testing, bioanalytical method validation, assay accuracy, assay precision

## Abstract

**Background/Objectives**: The quality of clinical studies is largely determined by the bioanalytical methods used for testing study samples. Rigorous assay validation following defined criteria, for example, the European Medicines Agency guideline for bioanalytical method validation, is a prerequisite for such assays. Alpha1-antitrypsin (AAT) measurement, i.e., the specific measurement of AAT protein and its associated elastase-inhibitory activity, is an integral part of assay panels for clinical studies addressing AAT deficiency. Specifically, AAT must be measured in the matrix of citrated human plasma as well as in diluted solutions with high salt concentrations obtained through bronchoalveolar lavage (BAL). Sensitive and selective measurement methods are required, as BAL has a low level of AAT. **Methods**: We present the validation data obtained for three AAT measurement methods. Two of them, nephelometry and the enzyme-linked immunosorbent assay, which clearly differ in their sensitivity, provide AAT protein concentrations. The third is the highly sensitive, newly developed elastase complex formation immunosorbent assay that specifically measures the inhibitory activity of AAT against its pivotal target, protease neutrophil elastase. Using samples with relevant AAT concentrations, we addressed the assays’ characteristics: accuracy, precision, linearity, selectivity, specificity, limit of quantification and short-term analyte stability **Results**: Overall, the three methods demonstrated low total errors, a combined measure reflecting accuracy and precision, even at low analyte concentrations of less than 0.5 µg/mL; adequate linearity over the required assay range; and acceptable selectivity and specificity. Furthermore, the short-time stability of the analyte was also demonstrated. **Conclusions**: All three AAT measurement methods met the acceptance criteria defined by the guidelines on bioanalytical assay validation, qualifying these methods for clinical sample analysis.

## 1. Introduction

Human alpha1-antitrypsin (AAT) [1], also known by its synonym alpha1-proteinase inhibitor, circulates at serum levels of 17 to 34 µM (0.9 to 1.75 g/L) with a half-life of 3–5 days [2]. The single chain, 52-kDa glycoprotein, containing 394 amino acids, is decorated with three complex type N glycans, on asparagines 46, 83 and 247. N-glycan branching and associated sialylation, as well as protein truncation have been described, as occurring at the protein’s N-terminus and C-terminus [3,4,5,6,7], resulting in a characteristic pattern of isoelectric focusing. AAT is a member of the serine proteinase inhibitor superfamily and is considered to be the prototype of this large family. After passive diffusion within the circulation, it is responsible for more than 90% of the anti-elastase activity of the lower respiratory tract.

The earliest evidence for protease inhibitory activity in serum/plasma dates back to the late 19th century [8,9]. Systematic studies on this trypsin-inhibitory activity contained in the α-globulin fraction of human serum [10,11] finally resulted in successful purification of trypsin-inhibiting activity from human plasma. The authors suggested naming the protein alpha1-antitrypsin [12].

Deficiency of this abundant α_1_-globulin was first detected by Laurell and Eriksson in 1963 [13,14]. They associated AAT deficiency (AATD) in five individuals with the early occurrence of emphysema, after having analyzed approximately 1500 serum samples with serum electrophoresis [15]. Owen et al. [16] and Jeppsson [17] set further landmarks in the history of AATD, because they described the amino acid substitutions E264V and E342K for the AATD-linked AAT variants. These AAT variants were later termed Pi*S and Pi*Z, respectively. Pi*ZZ AAT accounts for more than 96% of AATD cases.

Progress in nonclinical research enabled the first clinical trial, in which five Pi*ZZ patients were treated with a human plasma-derived AAT preparation [18]. The study was designed with the sole intention of showing the biochemical efficacy of what is today called AATD augmentation therapy [19,20,21]. Nevertheless, it specified the AAT dose and the dosing interval that are still used today. Ongoing clinical research using modern X-ray lung imaging techniques combined with advanced computational models has demonstrated the efficacy of AATD augmentation therapy [22,23,24]. To date, four plasma-derived AAT concentrates have received FDA approval. AATD patients are treated with a weekly intravenous infusion of 60 mg AAT per kg of body weight.

Clinical studies investigating the refinement of AAT augmentation or aiming to investigate new therapeutic applications of AAT in human diseases, including diabetes type-1 [25] and graft-versus-host disease [26], require measurement of the AAT activity and AAT protein concentrations in biological specimens. So-called bioanalytical methods are required for these analyses. This term [27,28], coined in the 1970s, covers the analytical methods that are used to determine the concentrations of drugs or their metabolites in biological fluids: blood, serum, saliva, urine, BAL and sputum, in addition to other tissues.

Parallel to the development of bioanalytical methods predominantly used to learn about the pharmacokinetic features of drug candidates or drugs, discussions on their performance characterization and associated regulatory topics took place [29]. One of the first guidance documents on bioanalysis was published by the FDA as part of the “Bioequivalence and Bioavailability” regulations in 1977 [30]. Discussions on bioanalytical criteria between scientists from the pharmaceutical industry and regulatory agencies were initiated. The findings of a meeting, titled “Analytical methods validation, bioavailability, bioequivalence and pharmacokinetic studies”, were summarized in a conference report [31]. Subsequently, the FDA published the “Draft guidance for industry: Bioanalytical method validation” in 1999. Further discussion focused especially on the validation acceptance criteria for immunoassays [32]. Then, the FDA issued its “Guidance for industry on bioanalytical method validation” in 2001. This document covered primarily the quantification of small molecules with chromatography, but has also been applied to large molecules and a variety of analytical techniques.

The EMA guideline, issued in 2011 [33], and the FDA guidance for industry, finally released in 2018 [34], provided the regulatory framework for bioanalysis. Both documents, however, were superseded by the ICH guideline M10 on bioanalytical method validation, which became effective in May 2022 [35]. The M10 guideline followed the acceptance criteria defined by the two previous documents. Furthermore, the new M10 guideline discriminated between ligand-binding assays (LBAs) and others. This discrimination highlights the predominant role of LBAs in bioanalysis with respect to measuring protein concentrations, as most of the pharmacokinetic data for therapeutic proteins were obtained by using LBAs [36].

The analysis of AAT-related samples from clinical studies likewise relies on the use of LBAs. Borja Ruiz-Duque et al. provide a comprehensive and systematic overview of the methodologies used for the determination of blood AAT levels [37]. Apart from measurement of AAT protein levels using immunological methods such as nephelometry or the enzyme-linked immunosorbent assay (ELISA), determination of functional AAT activity, expressed as (neutrophil) elastase-inhibitory activity, is also of interest. This can be achieved by using an elastase inhibition assay, which measures residual elastase activity in AAT samples that have been incubated with an excess of elastase. Specifically, N-succinyl-(L-alanyl)_3_-*p*-nitroanilide, a chromogenic substrate with selectivity for elastase, is used [37]. Standard assay formats using this chromogenic approach have a quantification limit of about 3–10 µg active AAT/mL. From the perspective of bioanalytical validation, this activity assay is classified as an LBA due to the involvement of binding reactions.

In addition to measurements of functional AAT activity in citrated plasma samples, samples obtained by BAL are also of interest. Given that the transfer of AAT from the bloodstream to the lung relies solely on diffusion, BAL samples typically exhibit AAT levels close to or even below the lower level of quantification of elastase inhibition assays. This peculiarity presents a unique challenge for analytical support of such studies. The newly developed functional AAT activity assay, termed elastase complex formation immunosorbent assay (ECFISA) [38], addresses this challenge by demonstrating a 1000-fold increase in sensitivity compared to the traditional chromogenic assay.

Accurate quantification of serum biomarkers such as AAT plays a pivotal role not only in diagnosing inherited deficiencies but also in monitoring drug-induced inflammatory conditions and immune responses [39]. With the increasing clinical use of agents like sodium-glucose cotransporter-2 inhibitors and immune checkpoint inhibitors such as pembrolizumab, reports of adverse effects, including acute pancreatitis, have underscored the importance of reliable protein-level assessments to guide therapeutic decisions and predict patient outcomes [39]. Additionally, bioanalytical advancements have supported broader disease surveillance strategies, such as the use of antioxidants and protein aggregation inhibitors in the management of cataractogenesis. These developments collectively reflect a growing demand for precise, sensitive, and specific protein detection technologies in both diagnostic and therapeutic contexts. Furthermore, recent innovations in probe chemistry—such as the use of highly efficient nitroaryl groups for hypoxia imaging in tumor cells—highlight the ongoing evolution of bioanalytical platforms toward higher selectivity and functional integration [40]. Within this framework, a systematic comparison and validation of nephelometry, ELISA, and the novel ECFISA for AAT quantification addresses a critical methodological gap and holds translational significance for clinical diagnostics and drug safety monitoring.

Here, we present the bioanalytical validation data covering accuracy, precision, linearity, selectivity, specificity, limit of quantification, and short-term analyte stability obtained for three methods used for measuring AAT levels in human citrated plasma and BAL-mimicking samples. Specifically, functional AAT activity was determined with the ECFISA in both sample types. The AAT protein levels of citrated plasma and BAL samples were measured with nephelometry and the ELISA, respectively, depending on the expected concentrations. In summary, all three AAT measurement methods met the acceptance criteria defined by current guidelines on bioanalytical assay validation, qualifying them for clinical sample analysis.

## 2. Results

### 2.1. Assay Calibration Curves

**Figure 1:**
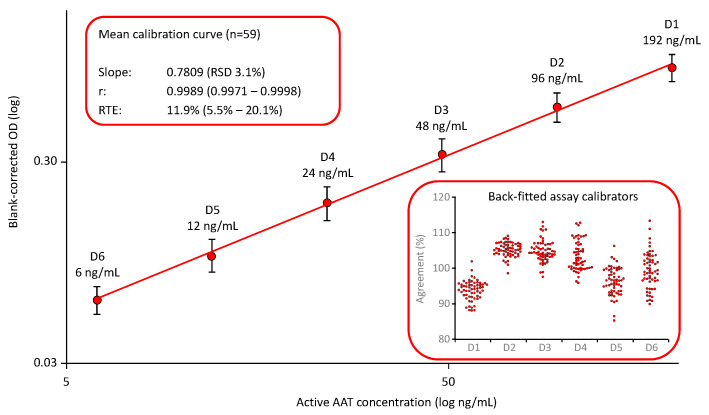
Mean calibration curve of the ECFISA AAT activity measurement. The mean (*n* = 59) six-point, log–log calibration ranging from 6 to 192 ng/mL functional active AAT/mL is shown. Error bars indicate the relative standard deviations of the means (red circles). The inserts provide mean quality characteristics including the slope, correlation coefficient and RTE of the calibrations curve and the agreement of the back-fitted assay calibrators with their nominal concentration, respectively.

**Figure 2:**
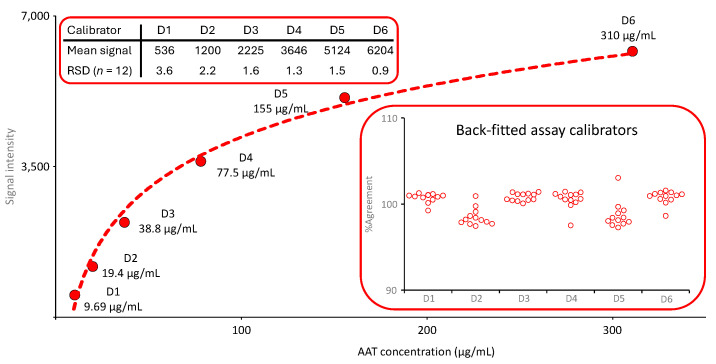
Mean calibration curve of the nephelometric AAT assay. The mean six-point calibration curve (*n* = 12) ranging from 9.69 to 310 µg AAT/mL is shown. Red circles indicate the mean signals. The inserts provide the mean signals obtained for the six assay calibrators and their RSDs and the agreement of the back-fitted assay calibrators with their nominal concentration, respectively.

**Figure 3:**
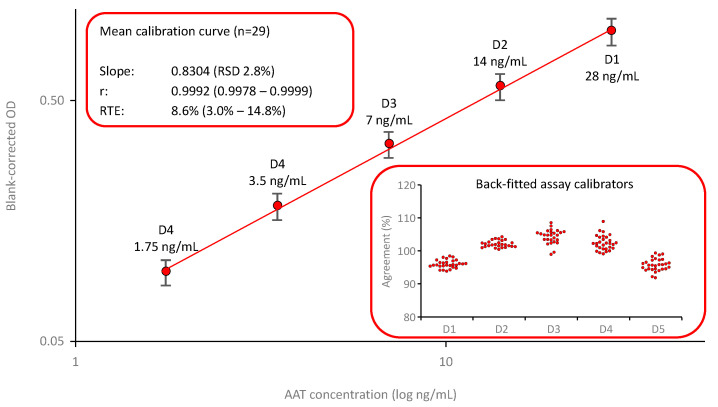
Mean calibration curve of the AAT ELISA. The mean (*n* = 29) five-point, log–log calibration ranging from 1.75 to 28 ng/mL AAT/mL is shown. Error bars indicate the relative standard deviations of the means (red circles). The inserts provide mean quality characteristics including the slope, correlation coefficient and RTE of the calibrations curve and the agreement of the back-fitted assay calibrators with their nominal concentration, respectively.

The mean calibration curves for the ECFISA, the AAT ELISA and the nephelometric AAT assay, are shown below (see Figure 1, Figure 2 and Figure 3). The calibration models for the ECFISA and ELISA calibration curves relied on linear regression of the (pseudo)linear part of the dose–response curves, obtained after log-transformation of both the blank-corrected optical densities (ODs) and the AAT concentrations of the assay calibrators. The calibration curve for the nephelometric assay is constructed by the nephelometer, based on a logarithmic regression analysis. No curve weighing was applied for any of the three calibration curves.

#### 2.1.1. AAT Activity Measurement with the ECFISA

Neither the EMA guideline for bioanalytical method validation nor the FDA guidance document provide preferences for the calibration curve fitting of LBAs. Based on our experiences with the development of the ELISA for measuring plasma proteins [41], we relied on a log–log fitting linear calibration model and defined a six-point calibration curve ranging from 6 to 192 ng functionally active AAT/mL. The AAT activity of the assay standard that we used to establish the calibration curve was assigned with reference to the first international WHO standard for AAT. Figure 1 shows the mean calibration curve obtained in 59 independent runs [38]. The mean slope showed a relative standard deviation (RSD) of 3.1%, demonstrating the low variability of these curves. Furthermore, the quality of curve fitting was reliable, as shown by the high correlation coefficients and the low relative total error values, with a mean relative total error (RTE) of 11.9%. The RTE was calculated as follows: The mean blank-corrected ODs of the individual assay calibrators D1 to D6 were back-fitted on the curve. The AAT levels obtained were then normalized by multiplication with their respective dilutions and averaged to obtain the measured mean concentration of the assay standard. Then, the absolute difference between the measured mean concentration of the assay standard and its nominal concentration was calculated and added to the double standard deviation determined for the mean. The sum obtained was then expressed as a percentage of the nominal concentration of the assay standard, in order to yield the RTE. The insert shows the good agreement of the back-fitted assay calibrators with their nominal concentrations: All individual data points were within a 100 ± 20% agreement, indicating a valid calibration curve.

#### 2.1.2. AAT Protein Measurement with Nephelometry

The calibration curve of the nephelometric AAT measurement was generated by the device, automatically checked for its quality and released for use before any sample analysis was carried out. The six-point logarithmic calibration curve covered an AAT range from 9.69 to 310 µg/mL. It was obtained by preparing a dilution series using the reference standard supplied with the kit, together with a labeled AAT concentration. Figure 2 shows the mean calibration curve obtained in 12 independent runs. The insert entitled “Back-fitted assay calibrators” presents the agreement of the back-fitted assay calibrators with their nominal concentrations. All individual values were within a 100 ± 5% range, demonstrating the adequate accuracy of the calibration curve fitting.

#### 2.1.3. AAT Protein Measurement with the ELISA

Figure 3 shows the mean calibration curve (*n* = 29) of the AAT ELISA, which was based on the use of a commercially available polyclonal anti-AAT capturing antibody and a polyclonal anti-AAT antibody peroxidase conjugate. The calibration curve was established by fitting the logarithms of the blank-corrected signals and the AAT concentrations of the assay calibrators. These calibrators, prepared by dilution of an in-house secondary reference standard that was calibrated against the international reference preparation ERM DA470k, covered a range from 1.75 to 28 ng AAT/mL. The calibration curves showed low variability, with an RSD of 2.8% for the mean slope. Furthermore, the quality of curve fitting was good, as shown by the high correlation coefficients and the low relative total errors, with a mean relative total error of 11.9%. The insert entitled “Back-fitted assay calibrators” shows the satisfactory agreement of the back-fitted assay calibrators with their nominal concentrations: All individual data points were within a 100 ± 10% agreement, indicating a valid calibration curve.

### 2.2. Accuracy Investigation

**Figure 4:**
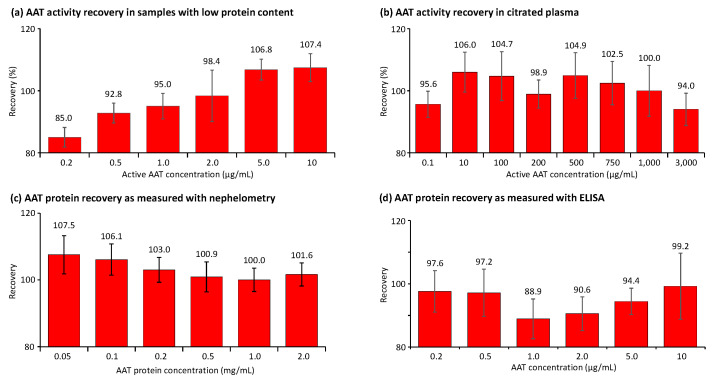
Recovery of AAT activity and AAT protein in samples with low protein content and citrated plasma. The recovery of AAT is shown as a percentage of the expected AAT concentration. (**a**,**b**) show the data obtained for the AAT activity measurement in BAL-mimicking samples with a low protein content and in citrated plasma, respectively, while (**c**,**d**) provide the recoveries of AAT protein, determined with the nephelometric method and the AAT ELISA.

The assays’ accuracy was investigated using several approaches. Figure 4 summarizes the recovery data obtained in the two sample types utilized for the validation, showing relevant concentrations of human AAT. First, validation samples with a human citrated plasma-like matrix were generated. These samples showed AAT concentrations ranging from 10 to 3000 µg AAT/mL and were obtained by either diluting the human normal plasma pool with 5% HSA or adding purified AAT. By contrast, samples with low protein content, mimicking samples obtained by BAL in terms of their AAT levels and salt concentrations, contained AAT concentrations from 0.2 to 10 µg/mL. The data shown represent the means of six independent measurements. Figure 4a,b show the recovery of AAT activity, as measured with the ECFISA in BAL-mimicking samples and human citrated plasma, respectively. All mean AAT activity recoveries were within an 85.0% to 107.4% range, thus complying with the 100 ± 20% acceptance range promoted by the EMA guideline and the FDA guidance document. As expected, we determined the lowest recovery, 85.0%, at the lowest AAT activity level, i.e., 0.2 µg/mL, which was associated with the sample with low protein content, while the recovery of 0.1 µg active AAT/mL in the citrated plasma was 95.6%, suggesting a general stabilizing effect of the plasma proteins. It should be noted, however, that such low AAT levels cannot be measured with conventional chromogenic or fluorogenic AAT elastase inhibition assays. In samples mimicking BAL fluid, the recovery of AAT activity exhibited a positive correlation, with increasing AAT concentrations ranging from 0.2 to 10 µg/mL. Conversely, this trend was not observed in citrated plasma samples. The mean recoveries of AAT protein, measured with nephelometry, in the citrated plasma samples containing 0.05 to 2 mg AAT/mL ranged from 100.0% to 107.5% (Figure 4c). For samples with low protein content and AAT protein levels ranging from 0.2 to 10 µg/mL, we found recoveries ranging from 88.9% to 99.2%, as measured with the ELISA (Figure 4d). In contrast to the activity data obtained for the same samples with the ECFISA, these recoveries did not correlate with the AAT protein levels.

Furthermore, we confirmed the accuracy of the AAT activity and the ELISA protein measurement by analyzing the first WHO standard for AAT with a labelled AAT concentration of 12.4 mg/mL. In doing so, we found a mean AAT activity and an AAT protein concentration of 98.0% ± 4.8% (*n* = 12) and of 100.1% ± 7.2% (*n* = 6), respectively. These results, showing good agreement with the labelled concentration, were obtained despite the highly diluted samples required for the measurement, due to the high sensitivity of both assays. Similarly, we measured the AAT protein concentration of the international standard ERM-DA470k with nephelometry. The mean AAT protein concentration (*n* = 6) that we found corresponded to 91.5% ± 6.5% of its defined AAT protein concentration. Thus, these data also demonstrated the adequate accuracy of the three tests.

Finally, the AAT activity concentrations, measured for 25 samples of purified AAT with a conventional validated chromogenic AAT activity assay and the ECFISA, were compared by means of a Bland–Altman plot [38,42]. The mean relative bias between the standard test and the ECFISA results was −0.64% ± 0.78%, resulting in 95% levels of agreement from −2.2% to 0.9%. This evidenced adequate agreement between the AAT activity measurement methods, with slightly higher values having been obtained for the ECFISA, which is remarkable, as we had to prepare highly diluted samples because of the ECFISA’s sensitivity.

### 2.3. Precision Investigation

**Figure 5:**
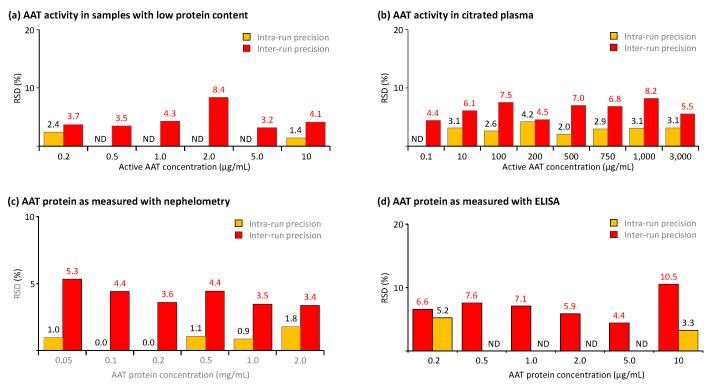
Results of the precision analysis. ND stands for not done, because intra-run precision was determined using a bracketing approach, if the analysis procedure included the measurement of a serial dilution series. The results of the precision analysis are expressed as the RSDs obtained for the means of six independent tests, carried out in one run (=intra-run precision) or six runs (=intermediate precision). (**a**,**b**) show the precision data of the AAT activity measurement in BAL-mimicking samples and citrated plasma, while (**c**,**d**) show the precision data of the AAT protein measurement with the nephelometric method and the ELISA, respectively, carried out for citrated plasma and BAL-mimicking samples.

We also used the samples prepared for the accuracy studies to determine the assays’ precision. Thus, we determined the methods’ precision profiles by measuring the intra-run (repeatability) and the intermediate precision, using, for each, six tests carried out in either one or six separate runs, the latter performed by different operators. Figure 5 summarizes the data obtained. Figure 5a,b show the precision of the activity measurement in samples with low protein content and citrated plasma, covering AAT activity ranges from 0.2 to 10 and from 0.1 to 3000 µg/mL, respectively, while Figure 5c,d present the precision data obtained for the AAT protein measurement with nephelometry in citrated plasma samples and ELISA in BAL-mimicking samples. Overall, for all three methods, we found RSDs not exceeding 10.5%, and thus clear compliance with the acceptance criteria of the EMA guideline and the FDA guidance, which require RSDs not exceeding 20% or 25% at the limit of quantification. In addition, the data did not identify any correlation between RSDs and analyte concentrations within the analyte ranges investigated. Remarkably, the three samples with AAT activity below 1 µg/mL showed RSDs not exceeding 4.4%, evidencing the excellent precision of the AAT activity measurement with the ECFISA, even for concentrations close to the assay’s lower limit of quantification. It seems reasonable to conclude that at all AAT levels and in both types of samples, intra-run precision was evidently better than intermediate precision.

Apart from these data, obtained during the assay validation study within a short period of time, we determined the AAT activity and AAT protein concentrations for the assay control, a lyophilized reference plasma preparation, using the three assays over an extended time of at least six months. Thus in 59 ECFISA runs, we found a mean ± SD AAT activity of 1046 ± 51.9 µg/mL, while the mean AAT protein concentrations, based on 29 runs, as determined with the nephelometric method and ELISA were 1090 ± 50 µg/mL and 996 ± 57.9 µg/mL, respectively. This translated to RSDs of 4.0%, 4.5% and 5.8%, respectively, which were within the same order of magnitude as those observed during the precision analysis carried out in six runs. These data also demonstrated the good precision of the three assays over an extended time.

### 2.4. Total Error and Quantitation Limits of the Assays

The overall assay performance characteristic total error (TE) was defined by the EMA guideline for bioanalytical method validation as the sum of accuracy and precision. It should not exceed 30% and 40%, respectively, at the assay’s lower limit of quantitation. Table 1 summarizes the total errors determined for the three methods at different AAT levels. TEs were calculated as the absolute difference of 100 and the recovery in %, added to the RSD, the latter of which described the intermediate precision. None of the total errors determined exceeded 18.7%. All thus met the acceptance criteria of the EMA guideline for valid assays. The AAT activity measurement in citrated plasma and BAL-mimicking samples demonstrated mean TEs of 10.0% (range: 5.6% to 12.2%) and 11.7% (range: 9.3% to 18.7%), respectively. The mean TEs determined for the nephelometric and the ELISA AAT protein measurement were 7.3% (range: 3.5% to 12.9%) and 12.3% (range: 8.9% to 18.1%), respectively. The quantitation limits of the ECFISA and the ELISA were 0.1 and 0.2 µg/mL, respectively, while the less sensitive nephelometric method had a limit of quantitation of 50 µg/mL.

### 2.5. Linearity Analysis Investigation

**Figure 6:**
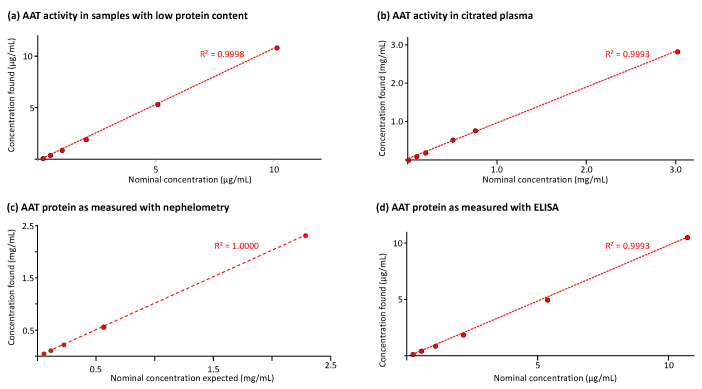
Results of the linearity analysis. The linear regression curves comparing the AAT concentrations as found with the nominal values are shown. The coefficient of determination R^2^ determined for these linear regression curves is also given. (**a**,**b**) show the curves for the AAT activity measurement in BAL-mimicking samples and citrated plasma, respectively; (**c**,**d**) show the regression curves obtained for the AAT protein measurement in citrated plasma and BAL-mimicking samples, respectively.

Figure 6 shows the results of the linearity analysis carried out for the three methods. Figure 6a,b provide the regression curves between the nominal AAT activity values and those found in samples with low protein content and citrated plasma, respectively. The AAT activity ranges covered by the regression curves were 0.2 to 10 µg/mL and 10 to 3000 µg/mL. Both regression curves had determination coefficients exceeding 0.999. Similarly, Figure 6c,d present the results of the AAT protein measurement with the nephelometric method in citrated plasma and the ELISA in samples with low protein content. The regression curves with determination coefficients higher than 0.999 ranged from 50 to 2000 µg/mL and from 0.2 to 10 µg/mL for the nephelometric and the ELISA method, respectively. These data demonstrated linearity. Furthermore, the ECFISA and ELISA dose–response curves obtained for the samples’ geometric 1 + 1 dilution series were checked for their correspondence with those of the respective assay calibration curves. The samples’ dose–response curves showed good linearity, with slopes that were highly similar to those of the respective calibration curves. Thus, no significant deviations from correspondence were found, confirming the absence of matrix effects.

### 2.6. Specificity and Selectivity Investigation

Specificity is the ability of a binding reagent to bind only to its target analyte. Selectivity describes how well an LBA quantifies the target analyte even when unrelated compounds are present. In this regard, we primarily investigated the AAT activity assay ECFISA for two reasons: to exclude unwanted interferences by other plasma protein inhibitors and to confirm its exclusive specificity for active AAT. In contrast to the ECFISA, which applies the formation of an elastase–AAT complex in order to measure the elastase-inhibitory activity of AAT, both AAT protein measurement methods are well established and are based on the use of commercially available antibodies against human AAT. These antibodies have a certified specificity for AAT, minimizing the importance of specificity/selectivity testing during the validation. Nevertheless, we checked the AAT ELISA for the possible influence of hyaluronic acid and human immunoglobulin G present in high excess over AAT.

The ECFISA detects the AAT–elastase complex formed using an AAT-specific antibody. Accordingly, we thought to rigorously rule out that functionally inactive AAT could be erroneously detected. Oxidation or moderate heat treatment was used to prepare functionally inactive AAT. As expected, analysis with a validated chromogenic elastase inhibition assay demonstrated that both preparations almost completely lost their inhibitory activity, without obvious effects on the AAT ELISA reactivity. This activity loss was also mirrored by the ECFISA (see Supplemental Materials). Moreover, we prepared mixtures of heat-treated and active AAT and analyzed them with the ECFISA. The data obtained clearly demonstrated that (i) the ECFISA measured functionally active AAT as intended and (ii) the presence of high excesses of functionally inactive AAT did not interfere with the measurement [38].

Human plasma contains several proteinase inhibitors which have been shown to form complexes with neutrophil elastase. Although the AAT–elastase complex formation rate constant is about 25-fold higher than those measured for other inhibitors, we nevertheless investigated whether or not the presence of the abundant plasma proteinase inhibitors alpha1-antichymotrypsin, alpha2-macroglobulin, alpha2-antiplasmin, antithrombin, C1-inhibitor and inter-alpha-trypsin inhibitor affected AAT activity measurement by the ECFISA in human plasma. All six inhibitors demonstrated only marginal, if any, complex formation with neutrophil elastase (see Supplemental Materials): maximum signals were found for alpha2-macroglobulin and alpha1-antichymotrypsin, counting for 6.0% and 3.9%, respectively, of that measured for AAT [38]. This allowed us to conclude that elastase complex formation with plasma inhibitors other than AAT does not interfere with the ECFISA by competing for the immobilized elastase. Moreover, these non-AAT–elastase complexes formed at a low abundance will not be detected, because the elastase–AAT complex is detected by using the specific anti-AAT antibody.

The AAT ELISA was checked for the possible influence of bovine serum albumin (BSA), hyaluronic acid and human immunoglobulin G. Accordingly, we measured samples with AAT concentrations close to the lower limit of quantitation (0.1 µg AAT/mL), and containing at least a 400-times excess of BSA, human immunoglobulin, or hyaluronic acid. The mean AAT recoveries (*n* = 6; mean ± SD) in these samples were 96.3 ± 6.0%, 93.0 ± 7.0% and 91.4 ± 4.9%, respectively, which ruled out the possibility that these compounds exerted substantial influence on the AAT ELISA, given their high excess over the analyte. Also, the dilution–response curves obtained for these samples closely paralleled that of the assay standard. The slopes of the spiked samples’ dilution curves differed by less than 10% from that of the respective assay standard measured on the same plate, thus demonstrating the absence of any matrix effect. Finally, a human purified-albumin preparation was shown to yield no signal in the nephelometric AAT protein assay.

### 2.7. Analyte Stability

#### 2.7.1. Short-Term Stability

We subjected the two sample types, citrated plasma and BAL-mimicking samples, to short-term stability studies, keeping them at room temperature (RT) for up to 48 h. The stability samples thus obtained were measured with the ECFISA, AAT ELISA and AAT nephelometry. AAT activity was measured in the citrated plasma samples containing 10 and 1000 µg active AAT/mL, and the BAL-mimicking samples with active AAT levels of 0.2 and 10 µg/mL. These samples, kept at RT for up to four hours, were also measured with the AAT ELISA. The samples analyzed with AAT nephelometry were kept at an even RT for 48 h. Table 2 summarizes the data obtained, expressed as a percentage of the respective initial concentration, as determined at the start of the study.

AAT activity and AAT protein were demonstrated to be stable at RT, i.e., to show changes of less than 20%, over four hours in citrated plasma as well as in BAL-mimicking low protein content samples. All but one of the relative concentrations measured differed by less than 10% from the initially determined ones. Consequently, we concluded that neither the AAT concentration nor the sample type had an influence on short-term stability. For the nephelometric AAT protein assay, we could even show the RT stability of the samples over 48 h.

#### 2.7.2. Stability on Repeated Freeze–Thaw Cycles

Repeated freezing–thawing of test samples can sometimes be required if only low quantities of clinical samples are available for analysis. We therefore investigated the effects of up to three repeated freeze–thaw cycles on AAT activity and AAT protein. Aliquots of 200 µL were frozen at −20 °C and kept at this temperature for 24 h. The samples were thawed by incubation at RT for 1 h. Table 3 presents the results of this study, which we carried out for the two sample types of citrated plasma and BAL-mimicking samples with low protein content.

All samples demonstrated acceptable stability, i.e., concentration changes of less than 20%, on repeated freezing–thawing, with the highest change in AAT activity being observed for the sample with the lowest AAT activity in the BAL-mimicking sample.

## 3. Discussion

The present work summarizes the results of the bioanalytical method validations of the three methods ECFISA, ELISA and nephelometry. In particular, ECFISA, a newly developed, highly sensitive assay, measures the inhibitory activity of AAT, while ELISA and nephelometry provide specific AAT protein concentrations. The validation study was performed as outlined by the EMA guideline. Thus, the assays’ characteristics, namely, accuracy, precision, linearity, selectivity, specificity, limit of quantitation, and the short-term stability of the analyte AAT, were addressed. The results obtained were in compliance with the requirements for bioanalytical methods.

The three methods validated here cover the specific AAT protein measurement and the determination of its inhibitory activity, i.e., its functionality. Functional AAT activity is usually measured with elastase inhibition assays that either use chromogenic or fluorogenic low-molecular weight peptide substrates or high-molecular weight substrates such as labeled elastins. In both cases, however, functional AAT activity is inversely proportional to the residual elastase activity measured with these assays. The basis for measuring functionally active AAT was laid down by the work of Bieth et al., who described the synthesis of the chromogenic substrate N-succinyl-(L-alanyl)_3_-p-nitroanilide, which shows good selectivity for elastase [43]. Standard assay formats using this substrate have a quantification limit of about 3–10 µg AAT/mL. This is adequate for the analysis of plasma/serum samples but does not allow direct measurement of AAT activity in BAL samples, in which concentrations of about 2 µg/mL of partially inactive AAT have been found [44]. Thus, a concentration step is required before AAT activity can be measured. Unfortunately, there is evidence that such a concentrating procedure could cause differential protein loss and functional AAT inactivation [45].

The ECFISA we validated here as an AAT activity assay does not rely on measuring the residual elastase activity, which is not inhibited by the action of AAT, but rather measures the amount of the complex formed between the two active reaction partners, AAT and elastase. The latter is immobilized by passive adsorption to the wells of a microplate and remains active, so complex formation with active AAT can take place. Sample components not involved in the inhibition reaction are removed by washing and the immobilized AAT-elastase complex is then immunologically detected by a specific anti-AAT peroxidase conjugate. This last assay step additionally increases the selectivity of the assay’s selectivity because only AAT–elastase complexes will be detected, even if other plasma protein inhibitor–elastase complexes are formed despite the high complex formation rate of the AAT–elastase complex. Rationally, other plasma protein inhibitors that are present in abundance, such as alpha2-macroglobulin, could compete for binding to the immobilized elastase and thus interfere with the assay. However, the selectivity study demonstrated that alpha2-macroglobulin-elastase complex formation had a negligible influence on the AAT activity measurement, as carried out in samples with plasma matrix. This contrasts with the chromogenic elastase inhibition assay, in which the presence of alpha2-macroglobulin erroneously increases AAT activity levels because alpha2-macroglobulin-bound elastase remains active when low molecular weight substrates are used for its measurement and cannot be inhibited by AAT due to steric hindrance. Similarly, the data confirmed that inactive AAT, generated either by heat treatment or oxidation of native AAT, did not interfere with the ECFISA AAT activity measurement.

The ECFISA’s major advantage over the chromogenic assay is its almost 1000-fold increased sensitivity. On the one hand, this increased sensitivity is reflected by the calibration curve which ranges down to single-digit ng/mL functional AAT levels. These curves could be obtained reproducibly with high accuracy and acceptable precision, as demonstrated by the good agreement of the back-fitted assay calibrators with their nominal concentrations. In addition, these curves were characterized by low relative total errors. On the other hand, the sample with an AAT activity of 0.2 µg/mL, representing the assay’s lower limit of quantification, was measured with acceptable accuracy and precision, resulting in a total error of 18.7%. This is clearly below the acceptance criterion of 40% required for valid LBAs. All other validation samples, irrespective of their type being either citrated plasma or BAL-mimicking samples, showed lower total errors, not exceeding 12.2%. Furthermore, there was no obvious correlation with the AAT activity levels. These low total errors mirror the assay’s good accuracy and precision in both sample types and over all the concentrations investigated. Thus, the mean RSDs, describing the assay’s intermediate precision, in six runs were 6.3% and 4.5% for the citrated plasma and BAL-mimicking samples, respectively. None of the RSDs exceeded 8.4%. The results obtained for the assay control over six months confirmed the short-term precision analysis data. Furthermore, repeated analysis of the first WHO standard for AAT resulted in a mean AAT activity that did not differ significantly from its labelled one, despite the high dilutions required to measure this sample. This result further highlights that the ECFISA method provides accurate AAT activity determinations. A direct comparison of AAT activity, as determined for 25 lots of a purified AAT preparation, utilizing a validated standard chromogenic test used for the release testing and the ECFISA, resulted in an absolute bias of 0.6%. Again, this confirmed the accuracy of AAT activity measurement with the ECFISA. Finally, adequate short-term stability for up to four hours at RT was shown for samples with relevant AAT levels in both matrices, citrated plasma and BAL-mimicking samples, and on repeated freezing–thawing. The results of long-term stability studies for samples kept over two years and measured with the ECFISA and the two other methods have recently been published [46].

The two other methods validated here, AAT nephelometry and the AAT ELISA, both measure AAT protein levels, and also follow the principles of LBAs. The measuring signal in the nephelometric assay, i.e., the turbidity of the solution caused by the antigen–antibody complex, is dependent on the binding to only one polyclonal antibody. The AAT ELISA is a conventional sandwich test: AAT, bound to the plate-immobilized polyclonal antibody, is then detected by the binding of a second polyclonal antibody labeled with peroxidase. The strict specificity of all test antibodies for human AAT is certified by their manufacturers. Both methods have clearly different sensitivities, as indicated by their calibration curves. These curves cover AAT levels in the low µg/mL (about 10 to 300) and the low ng/mL (about 2 to 30) ranges for the nephelometric assay and the ELISA, respectively. Rationally, this sensitivity profile qualifies the nephelometric method not only for the analysis of plasma samples obtained during the AAT augmentation therapy but also for identifying AAT-deficient patients. This conclusion is supported by a recent data analysis in which nephelometry was the most frequently used method for determining AAT blood levels, followed by ELISA [37].

Further advantages of the nephelometric method include the low operational effort required, because analysis is largely automated; the short time required to obtain results; and the automatic identification of samples, in which turbidity, lipidic material, or other effects could interfere with the AAT protein determination. The validation data obtained for AAT concentrations ranging from 50 to 3000 µg/mL demonstrated the adequate performance of the nephelometric method: Total errors did not exceed 12.9%, and the mean value was 7.3%. The mean intermediate precision was 4.1%. This good precision determined over a short period of time was confirmed by the data obtained over six months, in which the assay control showed an RSD of 4.5%. The AAT concentration determined for the international immunological reference preparation ERM DA470-k corresponded to 91.5% of its labelled AAT protein value, thus confirming the listed AAT concentration of the assay standard supplied with the nephelometric kit.

The ELISA method requires much more operational effort than the nephelometric method, and more time to obtain results. These disadvantages are counteracted by the method’s higher sensitivity. This sensitivity is clearly required for the analysis of BAL samples. Usually, BAL samples have total protein levels of less than 100 µg/mL. Albumin (~50% of total protein), the immunoglobulins G and A (together ~30%) and transferrin (~5%) account for almost 90% of the low total protein content [47,48]. Mean AAT levels of 1.8 and 3.1 µg/mL were reported for nonsmokers (*n* = 10) and smokers (*n* = 11), respectively [49]. Thus, AAT represents not more than 3% of the total protein present in BAL. Such low AAT concentrations can be accurately and precisely measured, as shown by the validation data obtained for samples with AAT concentrations ranging from 0.2 to 10 µg/mL. The mean total error was 12.4%, and none of the results exceeded 18.1%, which clearly meets the acceptance criterion requiring not higher than 30%. Interestingly, the highest total error was not determined for the lowest AAT concentration. The mean intermediate precision determined for these six BAL-mimicking samples was 7.0%, with all samples showing RSDs not exceeding 10.5%. Furthermore, we found the AAT protein concentration for the first WHO standard for AAT corresponded to 100.1% of its labelled concentration of 12.4 mg/mL. This shows that accurate and precise data can be obtained with the ELISA even for samples with such high AAT levels, despite the high dilutions required (the dilution series started at a dilution of 1/500,000). Interference by human immunoglobulin G and hyaluronic acid on the performance of the AAT ELISA could be ruled out by the data obtained in the spiking experiment.

A probable limitation of our validation studies is that we were unable to use human BAL samples. Such samples, however, can only be obtained under a clinical protocol, which requires validated methods. The BAL-mimicking samples that we prepared and used showed low total protein—mainly human albumin and AAT concentrations—thus resembling real-life BAL samples as closely as possible regarding these characteristics. Of course, human BAL samples will be used when available for a spike–recovery experiment to exclude any unexpected matrix effects. Another point to consider is that the AAT ELISA calibration curve comprises five points only, instead of six as promoted for LBAs. The AAT ELISA calibration curve, however, is not obtained by a 4/5-parameter fitting algorithm, as usually performed for ELISAs, but by a less complex linear regression analysis of the log-transformed, blank-corrected ODs and the AAT concentrations of the assay calibrators. This curve-fitting model, which was shown by the data from the back-fitting analysis to be highly accurate and precise, reduces the number of calibrators needed, as it relies only on the (pseudo)linear part of the dose–response curve. Furthermore, we learned in a small experiment that adding up to two additional calibration points without changing the AAT concentration range of the calibration curve had no effect on the characteristics of the calibration curve.

Overall, the data demonstrated that the three bioanalytical methods, elastase complex formation immunoassay, AAT nephelometry and AAT ELISA, were accurate, precise and selective methods for measuring functionally active AAT and AAT protein, even at low AAT concentrations. Such AAT concentrations were previously inaccessible to direct activity measurement without a preceding concentration step.

## 4. Materials and Methods

### 4.1. Materials

Chemicals from VWR (Vienna, Austria) were used: NaHCO_3_, Na_2_CO_3_, KCl, NaCl, KH_2_PO_4_, Na_2_HPO_4_ × 2 H_2_O, H_2_SO_4_ (95–97%), water (HPLC grade) and HCl (25%). Tween 20 (EIA grade) was obtained from Bio-Rad (Vienna, Austria); bovine serum albumin (BSA, A0281), porcine elastase (E7885) and benzamidine hydrochloride monohydrate (B6506) from Sigma (Vienna, Austria); sodium hyaluronate was acquired (part 81; Lifecore Biomedical, Chaska, MN, USA); non-fat dry milk was obtained from Maresi (Vienna, Austria); Patentblau V was acquired from Chroma-Waldeck (Münster, Germany); and the tetramethylbenzidine peroxidase substrate SureBlue was obtained from KPL (Medac; Hamburg, Germany). The citrated lyophilized human reference normal plasma pool, comprising plasma from at least 100 healthy donors, was purchased from Technoclone (Vienna, Austria). The purified human plasma proteins serum albumin (5%; #0100301A; HSA; purity > 95%), human alpha1-proteinase inhibitor (Aralast NP, 20 mg/mL; #VNB5K036) and the human immunoglobulin preparation (Gammagard Liquid, 100 mg/mL; #LE12E003) were from Takeda Manufacturing (Vienna, Austria). All other biological reagents and standards used are described with the respective methods (see Supplemental Materials).

### 4.2. Preparation of Validation Samples

We prepared two types of validation samples substantially differing in their matrices. For the first type, we either diluted the human reference plasma with the purified 5% human serum albumin preparation, or we added the purified human AAT preparation Aralast NP to obtain samples with AAT levels ranging from 0.01 to 3 mg AAT/mL. The second type of validation samples, designed to mimic samples obtained by the BAL procedure, showed low AAT levels ranging from 0.2 to 10 µg/mL in the presence of 0.9% NaCl and 50 µg/mL HSA. After preparation, samples were divided into aliquots of 500 µL, which were immediately frozen at –20 °C and stored frozen at –20 °C until analysis.

### 4.3. Performance of the Validation

Our validation studies addressed the methods’ characteristics: accuracy; precision, as intra- and intermediate precision as well as precision over an extended time; linearity; specificity; and selectivity. Furthermore, we investigated the stability of AAT activity and AAT protein at the bench at RT for up to 48 h and during up to three repeated freezing–thawing cycles. In particular, we determined the methods’ accuracy by evaluating the quality of the calibration models by spike–recovery studies and by measurement of international reference preparations, based on which, we determined the agreement of the AAT concentrations found with their labelled values. Intra- and intermediate precision were determined by performing six repetitions in either one or six different test units. The results for the assay controls were used to estimate the assay precision over an extended period. Selectivity and specificity were confirmed using the ECFISA by preparing and analyzing inhibitory inactive AAT, which was obtained by heat aggregation or oxidation. In addition, we checked the matrix of citrated plasma for the possible interfering influence of abundant human plasma proteinase inhibitors, including alpha2-macroglobulin, alpha1-antichymotrypsin, antithrombin, C1-inhibitor, inter-alpha-trypsin inhibitor and alpha2-antiplasmin, relative to the ECFISA. To detect the formation of the respective elastase–inhibitor complexes in the presence of plasma AAT levels, we used polyclonal antibody–peroxidase conjugates from The Binding Site (Thermo Fisher Scientific, Birmingham, UK: alpha2-macroglobulin: PP039; alpha1-antichymotrypsin: PP033; antithrombin: PP040; C1-inhibitor: PP019.X; inter-alpha-trypsin inhibitor: PP060; alpha2-antiplasmin: PC038; the latter peroxidase conjugate was prepared using the activated peroxidase from Roche [11428861001; Sigma, Vienna, Austria]). Functionally inactive AAT was prepared by heat treatment and oxidation using purified plasma-derived AAT Aralast NP. For the heat treatment, the reconstituted AAT was kept at 60 °C for 15 min, generating a maximum level of aggregated AAT. Oxidation with H_2_O_2_ was carried out as described [50].

### 4.4. AAT Measurement Methods

Functional AAT activity was determined with the elastase complex formation immunosorbent assay ECFISA [38], as recently described [46]. Briefly, we coated porcine elastase (E7885; Sigma, Vienna, Austria) on Maxisorp F96 plates. The AAT–elastase complex formed by functionally active AAT was detected using sheep anti-human AAT peroxidase (The Binding Site PP034, Thermo Fisher Scientific, Birmingham, UK). Bound peroxidase was measured with the peroxidase substrate SureBlue. The assay standard was calibrated against the WHO 1st international standard for alpha1-antitrypsin [51]. The AAT protein measuring methods, i.e., the AAT ELISA and the AAT nephelometric test, are also described in detail in [46]. Briefly, the AAT ELISA applied Maxisorp F96 plates and commercially available polyclonal antibodies: Rabbit anti-human AAT IgG A0012 (DakoCytomation A0012, Agilent, Glostrup, Denmark) as the capturing antibody and sheep anti-human AAT IgG peroxidase (The Binding Site PP034; Thermo Fisher Scientific, Birmingham, UK) as the detection antibody. The assay was calibrated using the international reference preparation ERM DA 470k [52]. For the nephelometric AAT protein determination, the clinical test available for the ProSpec BN nephelometer (Siemens, Vienna, Austria) was used. Detailed assay protocols for the three assays validated here can be found in [46] and in the Supplemental Materials. Finally, the AAT elastase-inhibitory activity of the purified plasma-derived AAT preparation Aralast NP was determined with a chromogenic elastase inhibition assay during its release testing, as described in [38]. The calibration curve of this chromogenic assay ranges from 4.4 to 11 µg functionally active AAT/mL. The AAT activity data obtained with the chromogenic and ECFISA methods were compared using the Bland–Altman method [42].

## Figures and Tables

**Table 1:** Total errors for the AAT activity and AAT protein measurement methods.

AAT Activity				AAT Protein			
ECFISA				Nephelometry		ELISA	
Citrated Plasma		BAL-Mimicking Sample		Citrated Plasma		BAL-Mimicking Sample	
µg/mL	TE ^(1)^ (%)	µg/mL	TE (%)	µg/mL	TE (%)	µg/mL	TE (%)
*0.1 ^(2)^*	*8.8*	*0.2*	*18.7*	*50*	*12.9*	*0.2*	*8.9*
10	12.1	0.5	10.7	100	10.5	0.5	10.4
100	12.2	1.0	9.3	200	6.6	1.0	18.1
200	5.6	2.0	10.0	500	5.3	2.0	15.3
500	11.9	5.0	9.9	1000	3.5	5.0	10.0
750	9.3	10	11.6	2000	5.0	10	11.3
1000	8.2						
3000	11.5						

^(1)^ TEs were calculated for each AAT concentration as the sum of the absolute difference of 100 and mean recovery and the RSD, describing the intermediate precision. ^(2)^ The data in italics give the results obtained at the limit of quantification.

**Table 2:** Short-term stability on the bench.

h at RT	%Initial AAT Concentration					
	Citrated Plasma		BAL-Mimicking Samples			
	AAT Activity (ECFISA)		AAT Activity (ECFISA)		AAT Protein (ELISA)	
	10 µg/mL	1000 µg/mL	0.2 µg/mL	10 µg/mL	0.2 µg/mL	10 µg/mL
1	102.7 ^(1)^	104.3	106.3	100.0	95.0	100.0
2	104.8	102.6	106.3	100.9	95.0	94.3
3	104.2	105.2	100.0	100.9	95.0	101.9
4	106.6	113.8	100.0	98.1	100.0	97.1
**h at RT**	**%initial AAT concentration**					
	**Citrated plasma**					
	**AAT protein (nephelometry)**					
	**50 µg/mL**	**500 µg/mL**	**1000 µg/mL**			
4	100.0	100.0	96.4			
24	97.0	98.3	94.5			
48	98.8	97.1	93.8			

^(1)^ The stability data are shown as a percentage of the initially measured AAT activity or AAT protein concentration. All results correspond to the means of triplicates, measured in one run. The RSDs of the means of the triplicates did not exceed 5%.

**Table 3:** Freezing–thawing stability.

Cycle	%Initial AAT Concentration								
	Citrated Plasma		BAL-Mimicking Samples				Citrated Plasma		
	ECFISA (µg/mL)		ECFISA (µg/mL)		ELISA (µg/mL)		Nephelometry (µg/mL)		
	10	1000	0.2	10	0.2	10	50	500	1000
1	96.9 ^(1)^	107.1	88.2	102.8	105.0	98.3	102.6	101.9	108.5
2	99.1	107.6	88.2	101.8	100.0	101.6	102.0	100.6	106.9
3	103.5	110.2	88.2	101.8	90.0	93.4	102.6	98.8	105.7

^(1)^ The stability data are shown as a percentage of the initial AAT activity or AAT protein concentration, as measured for the sample kept at RT for 0 h. All results correspond to the means of triplicates measured in one run. The RSDs of the means of the triplicates did not exceed 5%.

## Data Availability

The raw data supporting the conclusions of this article will be made available by the authors on request.

## Supplementary Materials

The following supporting information can be downloaded at: https://www.mdpi.com/article/10.3390/ph18081165/s1, S1. Test protocol for the ECFISA; S2. Test protocol for the AAT protein measurement with ELISA; S3. Test protocol for the nephelometric AAT protein measurement; S4. Bland-Altman comparison of AAT activity concentrations obtained with the conventional chromogenic and the ECFISA method; S5. Specificity of the ECFISA for native AAT; S6. Reactivity of other plasma proteinase inhibitors in the ECFISA.

## Abbreviations

The following abbreviations are used in this manuscript:

AAT	α1-antitrypsin
AATD	Alpha1-antitrypsin deficiency
BAL	Bronchoalveolar lavage
ECFISA	Elastase complex formation immunosorbent assay
ELISA	Enzyme-linked immunosorbent assay
EMA	European Medicines Agency
FDA	Food and Drug Administration
LBA	Ligand-binding assay
ND	Not done
OD	Optical density
RSD	Relative standard deviation
RT	Room temperature, 20–25 °C
RTE	Relative total error
SD	Standard deviation
TE	Total error
