# Supplementary material for: Bioanalytical Method Validations of Three Alpha1-Antitrypsin Measurement Methods Required for Clinical Sample Analysis
Source: Pharmaceuticals (Basel). 2025 Aug 6;18(8):1165. doi: 10.3390/ph18081165 (PMC12388989; doi:10.3390/ph18081165)
Supplement: Supplementary file 1 [file pharmaceuticals-18-01165-s001.zip › pharmaceuticals-3754224-supplementary.pdf]

S1 Test protocol for the ECFISA

The functional AAT measurement method uses solid phase-bound porcine elastase for determining the anti-elastase inhibition activity of AAT. The amounts of elastase-AAT complex formed are directly measured by detecting elastase-complexed, plate-bound AAT with a peroxidase-labelled anti-AAT antibody. The following materials were used: 96-well Maxisorp F96 flat bottom plates (Nunc, VWR; Vienna, Austria), polypropylene tubes (Greiner, Vienna, Austria), microtubes and disposable pipette tips (Bio-Rad, Vienna, Austria), parafilm and reagent reservoirs (Costar, Vienna, Austria). The following chemicals were from VWR (Vienna, Austria): KCl, NaCl, KH_2PO_4, $Na_2HPO_4 \times 2\ H_2O$, sulfuric acid (95-7%), water (HPLC grade) and HCl (25%). Bovine serum albumin (BSA, A0281) and porcine elastase (E7885) were from Sigma (Vienna, Austria), Tween 20 (EIA grade) from Bio-Rad (Vienna, Austria), Patentblau V from Chroma-Waldeck (Münster, Germany) and the tetramethylbenzidine substrate SureBlue from KPL (Medac, Hamburg, Germany).

The following buffers were used:

- ECFISA coating buffer (PBS): 8.0 g/L NaCl, 0.2 g/L KCl, 0.2 g/L KH_2PO_4, 1.26 g/L $Na_2HPO_4 \times 2\ H_2O$. Salts are dissolved in 1 L HPLC water; pH checked (target pH 7.2 ± 0.2) and 0.2 µm filtration with Nalgene filter unit (Sigma). The buffer can be stored at 4°C for two weeks.
- Washing buffer (PBST): PBS with 0.05% (v/v) Tween 20. This washing buffer can be stored at RT for one week.
- ECFISA dilution buffer (DB): 5 g BSA () are dissolved in 500 mL PBST. The DB used for the dilution of samples contains 0.025% (g/v) Patentblau V, added from a 2.5% aqueous stock solution. Dilution buffer has to be prepared freshly before use.
- Peroxidase substrate SureBlue (KPL) - ready to use.
- Stopping solution - 3 N sulfuric acid.

The following biological materials were used:

- Porcine elastase (Sigma, E7885).
- Sheep anti-human α_1-antitrypsin IgG peroxidase PP034 (The Binding Site, Thermo Fisher Scientific, Birmingham, UK).
- In-house AAT standard, 19.2 mg active AAT/mL, calibrated against the 1st WHO standard of human AAT, used as the assay calibrator.
- Human reference plasma 1A51 (Takeda, Vienna, Austria) as the control preparation.

Test sequence:

- *Coating*: Porcine elastase is dissolved in water (5 mg/mL) and kept frozen at -20°C in 50-µL aliquots for up to 12 months. Immediately before the plate coating, an aliquot is thawed and diluted 1/250 with ECFISA coating buffer. Nunc Maxisorp F96 plates are incubated with 100 µL/well coating solution at 4°C overnight.
- *Washing*: Coating is terminated by a washing step with washing buffer PBST done either manually or with a 96-well plate washer (Bio-Tek ELx-405). The washing is done three times; the emptied plate is then further processed.
- *Blocking of wells*: 200 µL/well ECFISA DB are added to the emptied wells using an 8-channel pipette or a dispenser (Multidrop 384 Dispenser). The plate is then incubated at 37°C for 60 min. Blocking is terminated by a single washing step. The emptied plate is then further processed.

- *Standard and sample dilution, loading and incubation*: Each well is filled with 100 µL/well DB ECFISA. Colored DB ECFISA is used for the dilution of standard and samples. The in-house assay standard with 19.2 mg AAT/mL is diluted 1/50,000, samples are diluted to obtain AAT concentrations of about 400 ng/mL. Serial 1+1 dilution series comprising six dilutions are then prepared directly on the plate by mixing 100 µL of the colored sample dilution with the dilution buffer in the well. Two independent dilution series are prepared. The samples loaded to row B are measured in only five dilutions as the positions B11 and B12 serve as blank and contain DB only. Fading of the color with progressing dilution from the left to right side of the plate reflects the serial dilution series prepared. Each plate contains dilution series for the assay standard, the assay control and six samples. The dilutions (100 µL/well) are then incubated at RT for 60 min. Sample incubation is terminated by three washing steps, followed by a further three washing steps after the plate has been rotated by 180 degrees. The emptied plate is then further processed.
- *Incubation with anti-AAT peroxidase:* Anti-human α_1-antitrypsin peroxidase (TBS PP034) is diluted 1/1,000 with DB. 100 µL/well are added and incubated at RT for 60 min. Incubation is terminated by a washing step, essentially carried out as described for the termination of the sample incubation.
- *Color reaction*: 100 µL/well SureBlue is added to the wells. The plate is incubated at RT for 15 min (protected for direct sunlight), before 100 µL/well stopping solution (3 N sulfuric acid) is added. Both additions can be done either manually or using the dispenser.
- *Plate measurement*: The plate is measured within 60 min with an ELISA reader (Bio-Tek EL-808) at 450 nm using a reference wavelength of 620 nm.
- *Data evaluation*: The calibration curve, ranging from 6 to 192 ng active AAT/mL, is obtained as a linear regression curve calculated for the blank-corrected mean ODs of the duplicates and the AAT concentrations of the six assay standards. For the sample evaluation, only ODs within the range defined by the calibration curve are considered. The concentrations obtained for the individual samples' dilutions are multiplied with the dilution and averaged to yield the final results.

S2 Test protocol for the AAT protein measurement with ELISA

The following materials were used for the AAT ELISA developed in-house: 96-well Maxisorp F96 flat bottom plates (Nunc, VWR; Vienna, Austria), polypropylene tubes (Greiner, Vienna, Austria), microtubes and disposable pipette tips (Bio-Rad, Vienna, Austria), parafilm and reagent reservoirs (Costar, Vienna, Austria). The following chemicals were from VWR (Vienna, Austria): Na_2CO_3, $NaHCO_3$, KCl, NaCl, KH_2PO_4, $Na_2HPO_4 \times 2\ H_2O$, sulfuric acid (95-7%), water (HPLC grade) and HCl (25%). Non-fat dry milk was obtained from Maresi (Vienna, Austria), benzamidine hydrochloride monohydrate from Sigma (Vienna, Austria), Tween 20 (EIA grade) from Bio-Rad (Vienna, Austria), Patentblau V from Chroma-Waldeck (Münster, Germany) and the tetramethylbenzidine substrate SureBlue from KPL (Medac, Hamburg, Germany).

The following buffers were used:

- Coating buffer: 0.1 M $NaHCO_3$, 0.1 M Na_2CO_3; dissolved in HPLC-grade water; pH 9.5 with HCl (25%)
- Washing buffer (PBST): phosphate-buffered saline (PBS), 0.8% NaCl, 0.02% KCl, 0.02% KH_2PO_4, 0.126% $Na_2HPO_4 \times 2\ H_2O$ with 0.05% Tween 20; pH 7.0-7.4.
- Blocking/dilution buffer (DB): 0.1% non-fat dry milk, 2 mM benzamidine in PBST.
- Peroxidase substrate SureBlue (KPL) - ready to use.

- Stopping solution: 1.5 M sulfuric acid.

The following biological materials were used:

- Rabbit anti-human α_1-antitrypsin IgG A0012 (DakoCytomation, Agilent, Glostrup, Denmark) as coating antibody.
- Sheep anti-human α_1-antitrypsin IgG peroxidase PP034 (The Binding Site, Thermo Fisher Scientific, Birmingham, UK) as detection antibody.
- Human serum calibrator ERM DA 470k with 1.12 mg AAT/mL as the assay calibrator.
- Human reference plasma 1A51 (Takeda, Vienna, Austria) as the control preparation.

Test sequence:

- *Coating*: The capturing antibody (rabbit anti-human α_1-antitrypsin IgG) was diluted to 10 µg/mL in coating buffer and 100 µL/well of this coating solution was incubated with a 96-well microplate at 4°C overnight.
- *Washing*: Coating is terminated by a washing step with PBST done either manually or with a 96-well plate washer (Bio-Tek ELx-405). The washing is done three times; the emptied plate is then further processed.
- *Blocking of wells*: 200 µL/well ECFISA DB are added to the emptied wells using an 8-channel pipette or a dispenser (Multidrop 384 Dispenser). The plate is then incubated at 37°C for 60 min. Blocking is terminated by a single washing step. The emptied plate is then further processed.
- *Standard and sample dilution, loading and incubation with detection antibody in SIMIT format:* Each well is filled with 100 µL/well DB. Colored DB is used for the dilution of standard and samples. The assay standard ERM DA 470k with 1.12 mg AAT/mL is diluted 1/40,000, samples are diluted to obtain AAT concentrations of about 60 ng/mL. Serial 1+1 dilution series comprising five dilutions are then prepared directly on the plate by mixing 100 µL of the colored sample dilution with the dilution buffer in the well. Two independent dilution series are prepared. The plate's positions A11 and A12 serve as blank and contain DB only. Fading of the color with progressing dilution from the left to right side of the plate reflects the serial dilution series prepared. Each plate contains dilution series for the assay standard, the assay control and six samples. The dilutions (100 µL/well) are then incubated at RT for 15 min. Then detection antibody, diluted 1/1,000 in DB is added (100 µL/well) and incubated at RT for 60 min. Incubation is terminated by three washing steps, followed by a further three washing steps after the plate has been rotated by 180 degrees. The emptied plate is then further processed.
- *Color reaction*: 100 µL/well SureBlue is added to the wells. The plate is incubated at RT for 15 min (protected for direct sunlight), before 100 µL/well stopping solution (3 N sulfuric acid) is added. Both additions can be done either manually or using the dispenser.
- *Plate measurement*: The plate is measured within 60 min with an ELISA reader (Bio-Tek EL-808) at 450 nm using a reference wavelength of 620 nm.
- *Data evaluation*: The calibration curve is obtained as a linear regression curve calculated for the blank-corrected mean ODs of the duplicates and the AAT concentrations of the assay standards. For the sample evaluation, only ODs within the range defined by the calibration curve are considered. The concentrations obtained for the individual samples' dilutions are multiplied with the dilution and averaged to yield the final results.

S3 Test protocol for the nephelometric AAT protein measurement

The nephelometer BN ProSpec (Siemens; Vienna, Austria) was used to measure human AAT protein in human citrated plasma samples. The reference standard and the reagents provided with the instrument were used. The nephelometer runs the following procedure: A reference curve with the N protein standard SL (Siemens, Vienna, Austria) is created per test unit. This standard with a defined, lot-specific AAT concentration is provided ready-to-use and stored at 4°C. The calibration curve obtained comprises the six dilutions 1/5, 1/10, 1/20, 1/40, 1/80 and 1/160 (0.298 to 0.009 mg AAT/mL). The calibration curve is automatically accepted if the mean deviation of the back-fitted concentrations of the calibration curve standards is ≤ 5%. In addition, the N/T protein control SL/M (Siemens, Vienna, Austria) is measured twice in independent dilutions. This control sample is also provided ready-to-use and is stored at 4°C. The AAT concentration determined must be within ± 15% of its labeled concentration for acceptance. In addition, the coagulation reference plasma pool standard 1R01 (Technoclone, Vienna, Austria) was established as an external assay control. This allowed us to run a control chart and check the inter-run assay precision independently of Siemens' reagents. The additional control sample was measured at least once per test unit in a 1/20-dilution using the N diluent provided by Siemens. For the sample analysis, the nephelometer automatically starts with a 1/20 dilution. If a sample shows an AAT concentration lower than the working range defined by the instrument, the sample is automatically re-analyzed using a 1/5 dilution.

S4 Bland-Altman comparison of AAT activity concentrations obtained with the conventional chromogenic and the ECFISA method

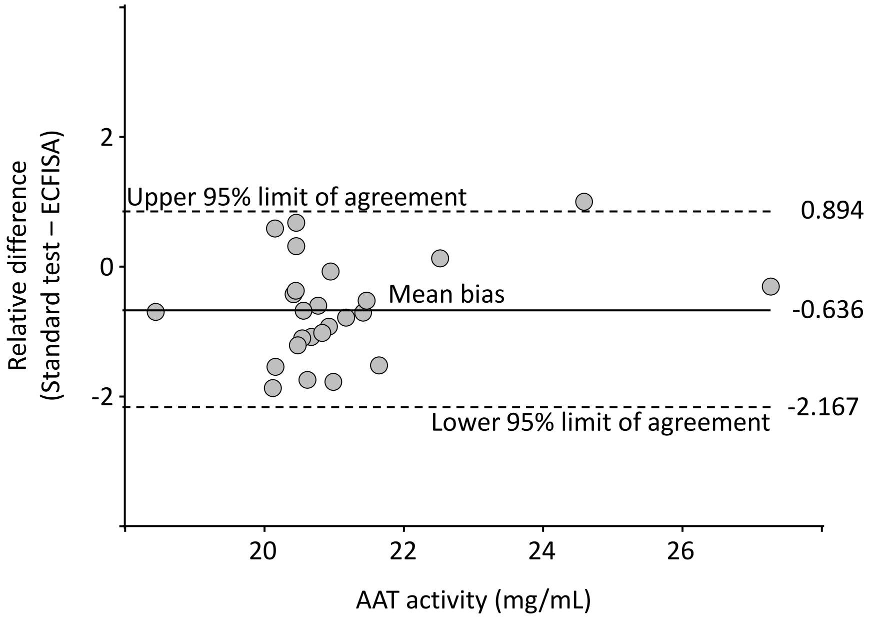

Bland-Altman comparison between the AAT activities measured with the conventional chromogenic test and the ECFISA was done for 25 lots of the highly purified AAT preparation Aralast NP.

S5 Specificity of the ECFISA for native AAT

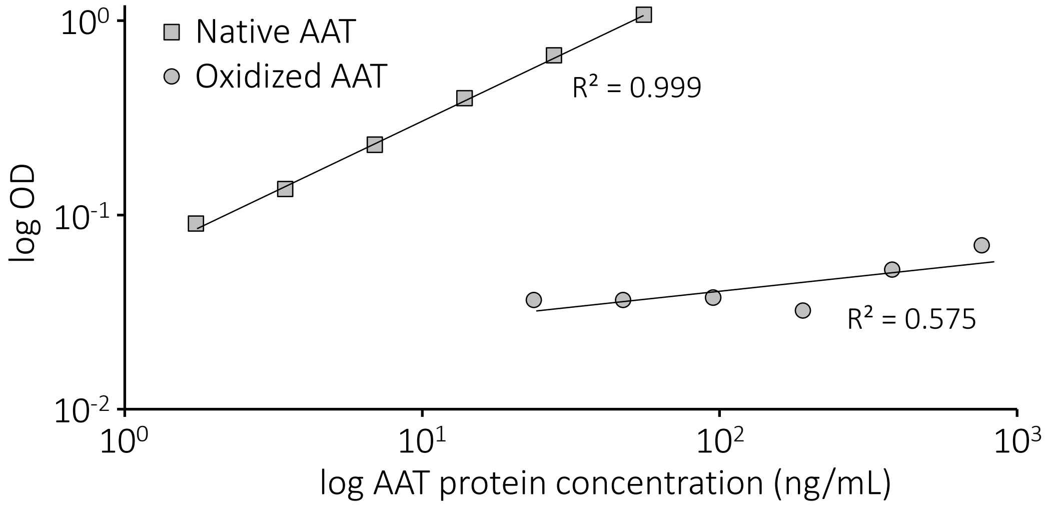

Oxidized AAT, showing less than 5% of its initial activity in the chromogenic standard AAT assay, demonstrated signals slightly above the blank when measured with the ECFISA. Please, note that data are shown in a double logarithmic plot.

S6 Reactivity of other plasma proteinase inhibitors in the ECFISA

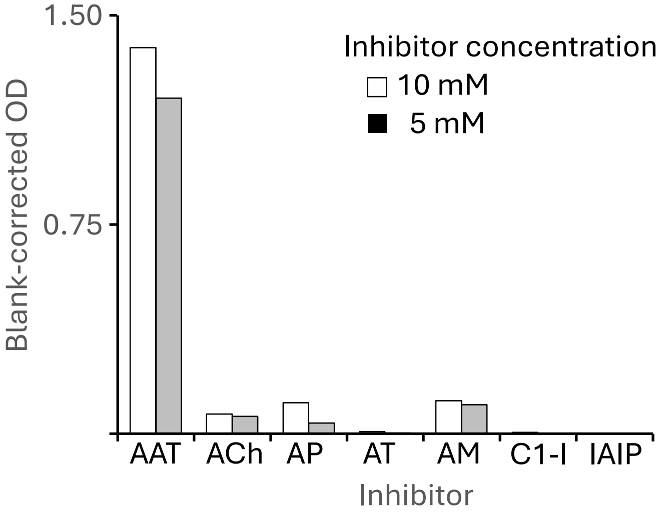

Low signals were observed when the elastase complexes formed with the plasma proteinase inhibitors alpha1-antichymotrypsin (Ach), alpha2-antiplasmin (AP), antithrombin (AT), alpha2-macroglobulin (AM), C1-inhibitor (C1-Inh) and inter-alpha-trypsin inhibitor (IAIP) were selectively detected after incubation of plasma.
